# The efficacy and safety comparison of first-line chemotherapeutic agents (high-dose methotrexate, doxorubicin, cisplatin, and ifosfamide) for osteosarcoma: a network meta-analysis

**DOI:** 10.1186/s13018-020-1576-0

**Published:** 2020-02-13

**Authors:** Bin Zhang, Yan Zhang, Rongzhen Li, Jiazhen Li, Xinchang Lu, Yi Zhang

**Affiliations:** 1 Department of Orthopedics, The First Affiliated Hospital of Zhengzhou University, No. 1 Jian she East Road, Erqi District, Zhengzhou City, 450052 Henan Province People’s Republic of China; 2 Department of Oncology, Sun Yat-Sen University Cancer Center, State Key Laboratory of Oncology in South China, Collaborative Innovation Center for Cancer Medicine, No. 651 Dongfeng East Road, Guangzhou, 510060 Guangdong People’s Republic of China

**Keywords:** Osteosarcoma, Chemotherapy, Methotrexate, Doxorubicin, Cisplatin, Ifosfamide, Network meta-analysis

## Abstract

**Background:**

Osteosarcoma, a primary malignant bone tumor derived from mesenchymal tissue, is the most common type of pleomorphic tumor that occurs in children and adolescents. The aim of this study was to compare the efficacy and safety of high-dose methotrexate (M), doxorubicin (D), cisplatin (C), and ifosfamide (I) in the management of osteosarcoma.

**Methods:**

Electronic databases including PubMed, Cochrane Library, and Embase database were searched for studies published from when the databases were established to July 13, 2019. The network meta-analysis was performed using software R 3.3.2 and STATA version 41.0 after demographic and outcome data extraction. The ranks based on probabilities of interventions for each outcome were performed. In addition, the consistency of direct and indirect evidence was assessed by node splitting.

**Results:**

The network meta-analysis results revealed that MDCI had a significant lower hazard risk of overall survival [MDCI vs MDC: HR = 0.74, 95% CrI (0.23, 0.87); MDCI vs DC: HR = 0.60, 95% CrI (0.16, 0.92)]. In addition, MDCI had a clearly longer progression-free survival time than that of DC [MDCI: HR = 0.88, 95% CrI (0.46, 0.98)]. No significant difference was detected in MDC and DC in OS, PFS, and AEs. The probabilities of rank plot showed that MDCI ranked first in OS (73.12%) and PFS (52.43%). DC was the best treatment in safety, ranked first (75.43%).

**Conclusions:**

MDCI showed its superiority among all chemotherapeutic agents in relation to efficacy and safety, followed by MDC. In addition, MDCI was associated with an increased risk of AEs. According to our analysis, DC was less effective but safer for MDC and MDCI.

## Background

Osteosarcoma, a primary malignant bone tumor derived from mesenchymal tissue, is the most common type of pleomorphic tumor that occurs in children and adolescents [1, 2]. Treatment of this bone cancer, the third most common malignancy in children and adolescents, was once mainly amputation, with very limited efficacy [3]. However, since the mid-1970s, multidrug chemotherapy, including high-dose methotrexate, doxorubicin, cisplatin, and ifosfamide, and surgical strategies have increased the 5-year overall survival rate of OS to 70–80% [4]. Nowadays, available several opinions are available for osteosarcoma, including systemic chemotherapy, targeted drug therapy, immunotherapy, radiotherapy, and experimental therapy [5]. Chemotherapy, a basic treatment, has been widely performed in clinical practice for many years. The common chemotherapy agents for osteosarcoma include high-dose methotrexate, doxorubicin, cisplatin, ifosfamide, gemcitabine, decitabine, etoposide, and vincristine [6, 7].

The mainstream chemotherapeutic agents include methotrexate, doxorubicin, cisplatin, and ifosfamide, which were the first-line chemotherapy agents approved by the NCCN Guidelines [8]. Since the introduction of chemotherapy, single-agent treatment of osteosarcoma is considered inadequate [9–11]. The majority of osteosarcoma treatment and research protocols is based on a combination of two or more of only four drugs: doxorubicin, cisplatin, methotrexate, and ifosfamide [12]. Therefore, eligible studies that compared doxorubicin + cisplatin (DC), methotrexate + doxorubicin + cisplatin (MDC), and methotrexate + doxorubicin + cisplatin + ifosfamide (MDCI) for osteosarcoma treatment were meaningful for our research. These three chemotherapy regimens recommended by the NCCN Guidelines Version 1.2020 have been the commonly used chemotherapy treatment for osteosarcoma patients [13].

Even though all preferred regimens have been widely used for many years, we still do not know the absolute role of each component of multidrug chemotherapy [14]. Therefore, which one is the optimal choice for use in osteosarcoma patients has still not been established. Also, it is difficult to say which chemotherapeutic regimen is superior. Furthermore, no systematic review and meta-analysis that accurately compared these three chemotherapy regimens including all the above four agents [15]. By adopting the new technology of network meta-analysis, we can achieve detailed comparisons, with direct and indirect evidence. Therefore, in the present protocol of network meta-analysis, we will aim to evaluate the efficacy and safety of the first-line chemotherapeutic agents for the treatment of osteosarcoma.

## Methods

### Searches

This network meta-analysis was performed in accordance with the guideline of Preferred Reporting Items for Systematic Reviews and Meta Analyses. We conducted a search of PubMed, Cochrane Library, and Embase databases from inception to July 2019. The Mesh terms and related synonym included “Osteosarcoma,” “OS,” “Osteosarcomas,” “Osteosarcoma Tumor,” “chemotherapy,” “Doxorubicin,” “Methotrexate,” “Ifosfamide,” “Cisplatin,” “randomized controlled trial,” “RCT,” and “randomly” were combined in the search strategy. We also manually searched the reference lists of related publications such as reviews and meta-analyses. The eligibility of the results retrieved was carefully examined with the use of EndNote software. Irrelative publications were removed by scanning the title, abstract, as well as the full texts.

### Inclusion criteria

In general, trials were considered eligible when they met the following criteria: (1) randomized controlled trials (RCTs), (2) all patients were diagnosed with osteosarcoma, and (3) two or more interventions among DC, MDC, and MDCI were compared.

### Exclusion criteria

The following criteria were used for exclusion: (1) other therapies were included, such as related receptor targeted therapy, immunotherapy, vaccine therapy, and radiotherapy; (2) insufficient data relating to treatments, outcomes of patients, and study design; (3) outcome data were unable to combined with other studies; and (4) letters, case reports, comments, meta-analysis, review and meeting abstracts, animal experiment, and basic research.

### Quality assessment

Two independent reviewers assessed methodological quality with Cochrane Handbook for Systematic Reviews Interventions version 5.1.3. For every included trial, the following criterions were evaluated and given a grade of low, medium, or high risk bias: random sequence generation, allocation concealment, blinding of participants and personnel, blinding of outcome assessment, incomplete outcome data, selective reporting, and other biases. Any disagreements on risk of bias ratings were regularly resolved through discussion by the two reviewers or by a consultation with a third reviewer.

### Data extraction

Two investigators performed the data extraction from qualified studies independently. The extracted data were as follows: first author’s name, country, journal, publication year, trail name, total study sample size, sample sizes of each treatment group, follow-up duration, and relevant clinical outcomes. The main outcomes included overall survival (OS), progression-free survival (PFS), and severe adverse events (AEs). OS was defined as the time from random assignment to death. PFS was defined as the time from randomization to first appearance of radiographic or clinical progression or death. Severe AEs were defined as grades ≥ 3. We analyzed the total number of severe AEs and the most common reported events including leukopenia, thrombocytopenia, red blood cell (RBC) transfusion, platelet (PLT) transfusion, granulocyte colony-stimulating factor (G-CSF) transfusion, and hospitalization.

### Data synthesis and analysis

The network meta-analysis was performed for each clinical outcome using R 3.3.2 software and STATA version 14.0 (Stata Corp LP, College Station, TX). The OS and PFS were treated as time-to-event variables, thus, these parameters were expressed as hazard ratio (HR) with 95% credible intervals (CI) for each study. In some studies, the HR with 95% CrI was not given directly but was presented as Kaplan-Meier curves; we calculated the values using the software of Engauge Digitizer 9.6 and the method provided by Tierney et al. [16]. If the survival curve was unavailable too, we estimated these values with the formulation log (HR) = (T1 + T2)2/[(E1 + E2)T1T2]. E1 and E2 are the numbers of events and T1 and T2 are the numbers of patients randomly assigned in each group [17]. Odds ratio (OR) with 95% CrI were calculated as the summary statistic for dichotomous variables such as leukopenia, thrombocytopenia, RBC transfusion, PLT transfusion, G-CSF transfusion, and hospitalization.

A random-effects model was utilized in this work to calculate evidence inconsistency as it was believed to be the most suitable and conservative method by which to speculate heterogeneity of various trials within each intervention comparison. The relative ranking of different outcomes was presented as the probabilities. In addition, publication bias was evaluated via observing the symmetry characteristics shown in funnel plots. A symmetrical and concentrated distribution of dots indicates no obvious deviation.

## Results

### Baseline characteristics of included studies

**Fig. 1:**
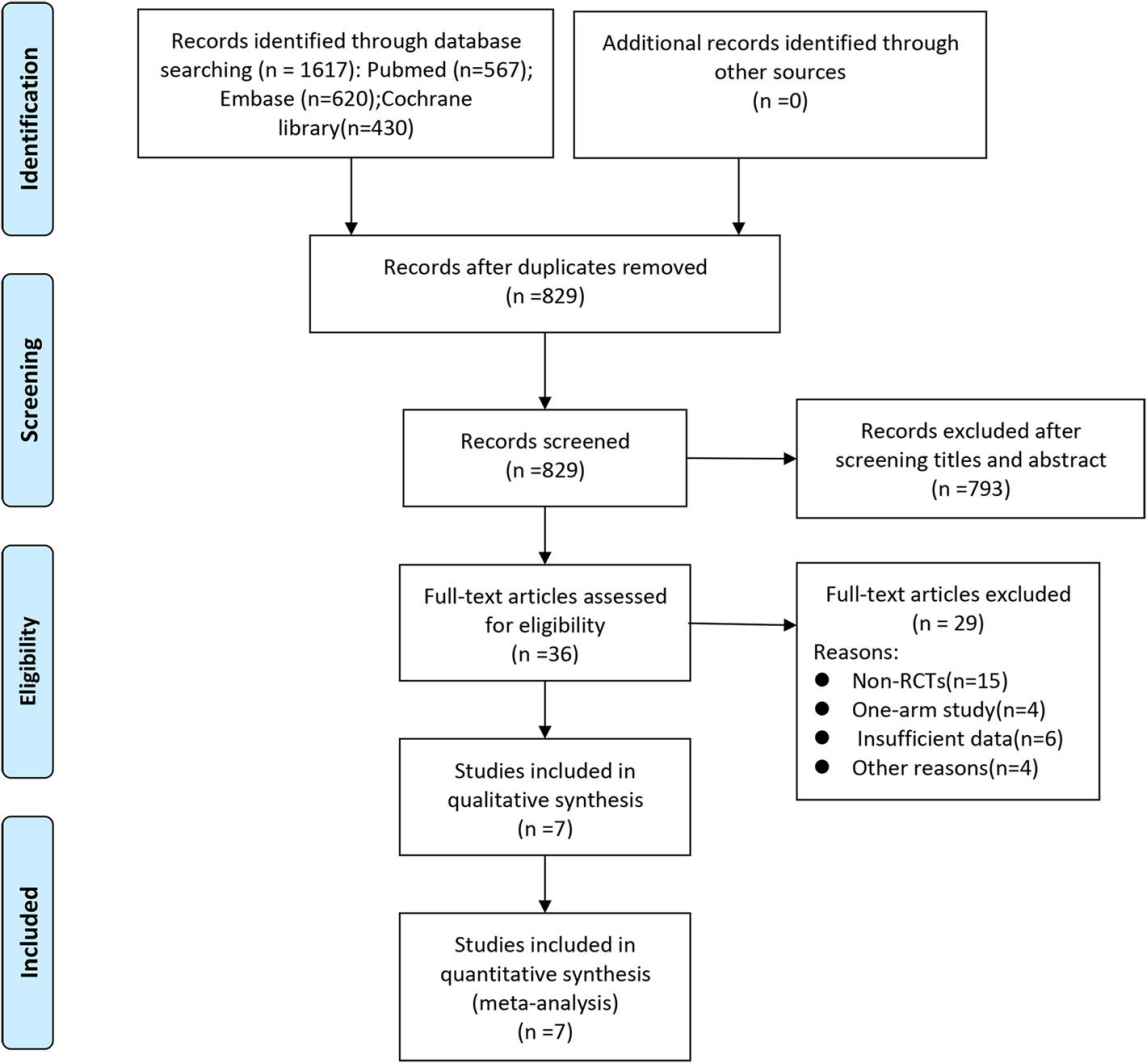
PRISMA flow diagram of the study selection process for the network meta-analysis

The entire process of searching and screening literature was presented in Fig. 1. A total of 1617 potential articles were identified from the databases search. After removing duplicates, 829 publications were screened by reading titles and abstracts. And 36 studies were performed a full-text assessment. Finally, 7 RCTs [18–24] with 1532 patients were enrolled into our analysis. The baseline characteristics of the 7 included studies were presented in Table 1.

**Table 1:** Characteristics of the included randomized controlled trials in network meta-analysis

Authors, year	Country	Study name, design	Experiment group regimens	Control group regimens	Samples (E/C)	Main outcomes
Craft et al. 2009 [22]	UK	EOI(80861), RCT	MTX 8 g/m^2^ DOX 25 mg/m^2^ daily times 3, CDP 100 mg/m^2^ 24-h infusion	DOX 25 mg/m^2^ daily times 3, CDP 100 mg/m^2^ 24-h infusion	191/197	OS, PFS, severe AEs
Whelan et al. 2012 [24]	UK	EOI(80931), RCT	MTX 8 g/m^2^ DOX 75 mg/m^2^ CDP 100 mg/m^2^ every 3 weeks	DOX 75 mg/m^2^ CDP 100 mg/m^2^ every 3 weeks^,^	90/89	OS, PFS, severe AEs
Ferrari et al. 2012 [23]	Italy	ISG/OS-1, RCT	MTX 12 g/m^2^ DOX 75 mg/m^2^ CDP 120 mg/m^2^ IFO 10 g/m^2^every 3 weeks	MTX 12 g/m^2^ DOX 75 mg/m^2^ CDP 120 mg/m^2^ every 3 weeks^,^	123/123	OS, PFS, severe AEs
Bramwell et al.1997 [20]	Canada	RCT	MTX 8 g/m^2^ DOX 25 mg/m^2^ CDP 100 mg/m^2^ every 3 weeks	DOX 25 mg/m^2^ CDP 100 mg/m^2^ every 3 weeks^,^	13/24	OS, PFS, severe AEs
Link et al. 1991 [18]	USA	MIOS, RCT	MTX 12 g/m^2^ DOX 75 mg/m^2^ CDP 120 mg/m^2^ IFO 10 g/m^2^every 3 weeks	MTX 12 g/m^2^ DOX 75 mg/m^2^ CDP 120 mg/m^2^ every 3 weeks	18/18	OS, PFS, severe AEs
Bramwell et al. 1992 [19]	Canada	RCT	MTX 8 g/m^2^ DOX 25 mg/m^2^ CDP 100 mg/m^2^ every 3 weeks	DOX 25 mg/m^2^ CDP 100 mg/m^2^ every 3 weeks	152/155	OS, PFS, severe AEs
Meyers et al. 2005 [21]	USA	CCG-7921, RCT	MTX 12 g/m^2^ DOX 75 mg/m^2^ CDP 120 mg/m^2^ IFO 9 g/m^2^every 3 weeks	MTX 12 g/m^2^ DOX 75 mg/m^2^ CDP 120 mg/m^2^ every 3 weeks	167/172	OS, PFS, severe AEs

*RCT* randomized controlled trial, *MTX* methotrexate, *DOX* doxorubicin, *CDP* cisplatin, *IFO* ifosfamide, *OS* overall survival, *PFS* progression-free survival, *AEs* adverse events

**Fig. 2:**
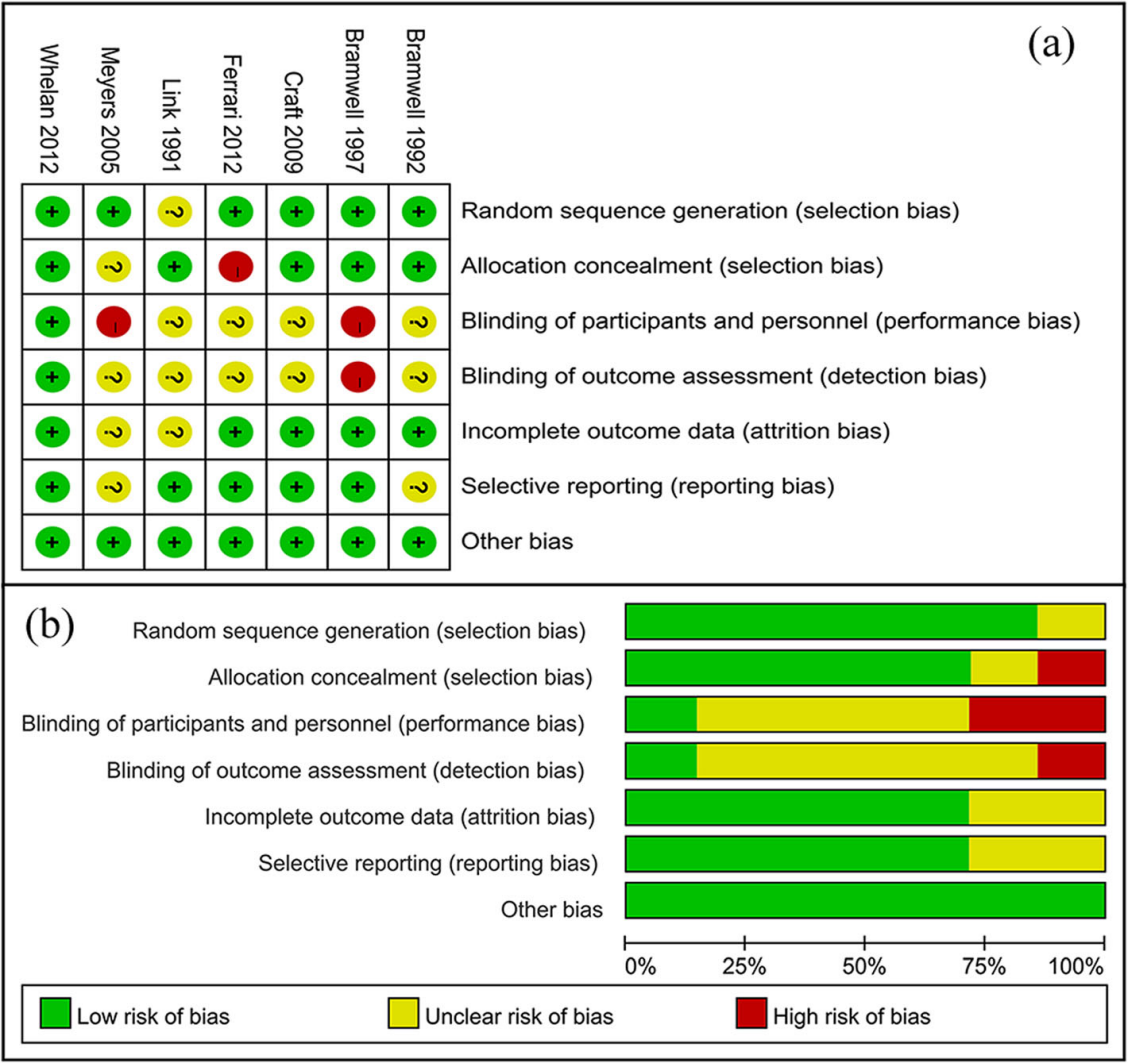
Risk of bias: graph and summary of the included studies. **a** Reviewers’ judgments of each risk of bias item for eligible studies. **b** The judgments of each item of risk of bias, presented as percentages across all eligible studies

The qualities of the eligible studies were assessed according to the Cochrane Collaboration tool for risk of bias assessment. All 7 included studies had strict randomization. Six out of the 7 eligible studies described the randomization process adequately, while the remainder did not explicitly describe the method of sequence generation. Five articles showed detailed information on allocation sequence concealment and the others did not. Meyers et al. have a sophisticated blind design. In the remaining studies, blinding of participants, personnel, and outcome assessment were not clearly stated. In short, most studies had a low risk of attrition and reporting bias. The risk of bias assessment of the eligible RCTs is summarized in Fig. 2.

### Network meta-analysis of overall survival

**Fig. 3:**
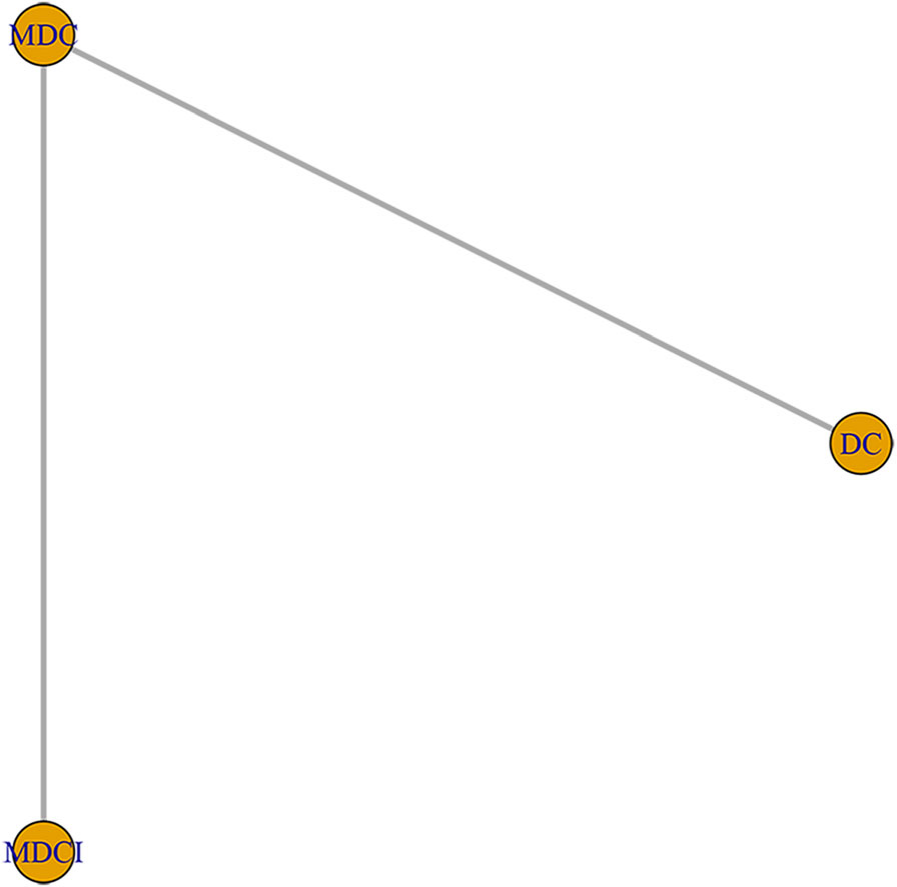
Network geometry of three chemotherapy regimens

**Fig. 4:**
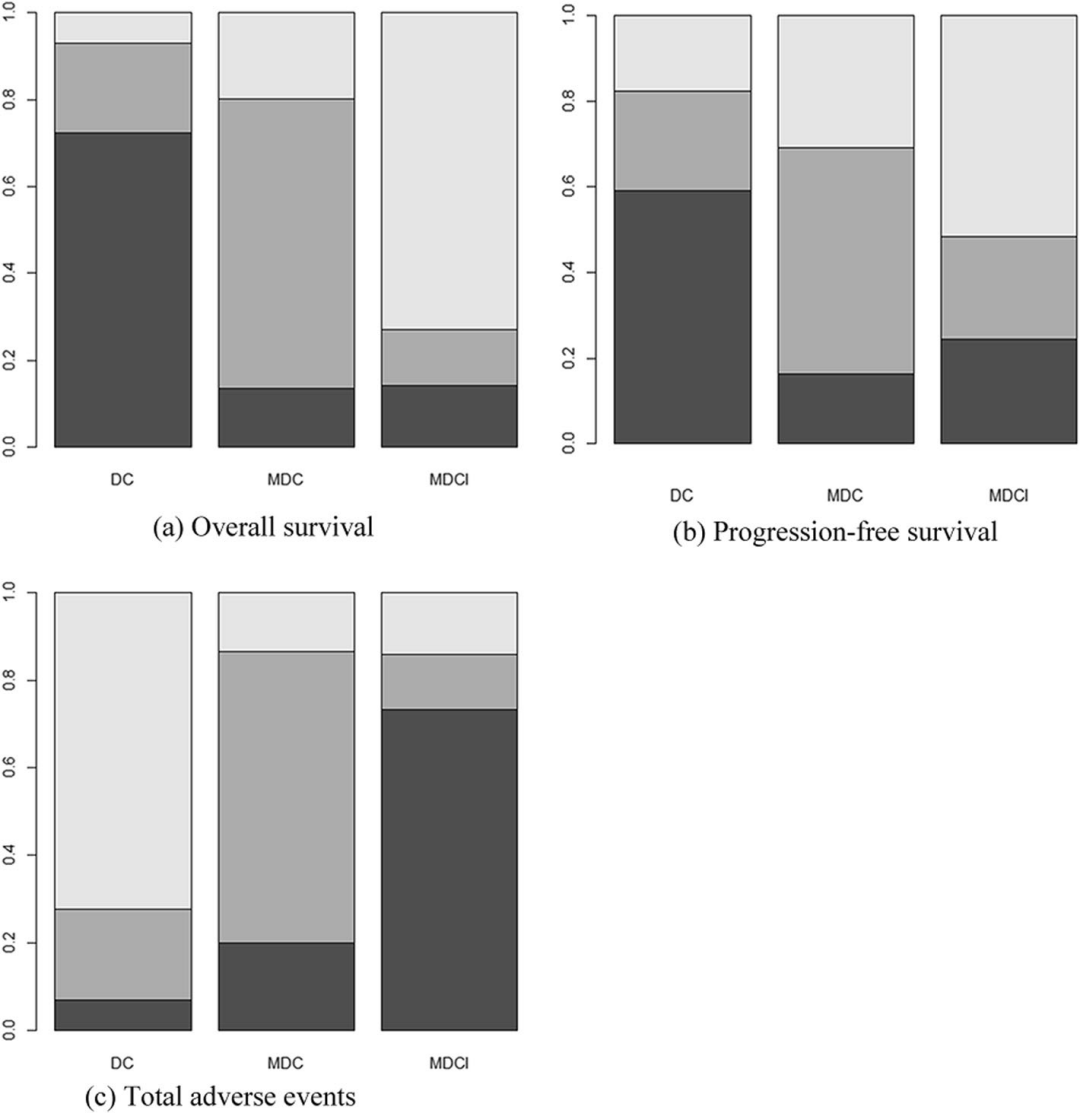
The relevant rank plots based on probabilities of interventions. **a** Overall survival. **b** Progression-free survival. **c** Total severe adverse events

All studies with 1532 patients provided the HR value or Kaplan-Meier curves for the OS. The direct comparisons between DC, MDC, and MDCI were shown by network plot (Fig. 3). The network meta-analysis results were presented in Table 2. Compared with MDC and DC treatment, MDCI had a significant lower hazard risk of overall survival [MDCI vs MDC: HR = 0.74, 95% CrI (0.23, 0.87), MDCI vs DC: HR = 0.60, 95% CrI (0.16, 0.92)], which meant that MDCI had a longer overall survival time than MDC and DC. For some reason, however, there is no statistically significant between DC and MDC. Meanwhile, probabilities of rank plot (Fig. 4) showed that MDCI ranked first (73.12%), MDC ranked second (67.41%), and DC ranked third (72.23%). Among the interventions, ranked first was the best treatment and the last was worst. The results of ranking analysis were illustrated in Fig. 4 a and Table 3.

**Table 2:** The network meta-analysis results of all outcomes

Outcomes	DC	MDC	MDCI
Overall survival (HR (95% CrI))			
DC	1	1.20 (0.71, 2.43)	1.71 (1.47, 6.42)
MDC	0.81 (0.42, 1.44)	1	1.32 (1.04, 4.35)
MDCI	0.60 (0.16, 0.92)	0.74 (0.23, 0.87)	1
Progression-free survival (HR (95% CrI))			
DC	1	1.12 (0.74, 1.75)	1.11 (0.63, 2.2)
MDC	0.91 (0.60, 1.45)	1	1.04 (0.67, 1.7)
MDCI	0.88 (0.46, 0.98)	0.97 (0.59, 1.51)	1
Total severe adverse events (OR (95% CrI))			
DC	1	0.56 (0.42, 0.76)	0.73 (0.44, 0.97)
MDC	1.17 (0.64, 2.10)	1	0.82 (0.42, 1.64)
MDCI	4.69 (2.79, 7.87)	2.64 (1.73, 4.05)	1
Leukopenia (OR (95% CrI))			
DC	1	0.45 (0.32, 0.74)	0.85 (0.54, 1.27)
MDC	1.25 (0.64, 3.15)	1	0.52 (0.12, 0.85)
MDCI	6.69 (4.79, 9.87)	2.14 (1.33, 4.45)	1
Thrombocytopenia (OR (95% CrI))			
DC	1	0.76 (0.34, 0.89)	0.72 (0.41, 1.17)
MDC	2.74 (1.64, 3.10)	1	0.78 (0.39, 1.53)
MDCI	3.14 (2.65, 7.75)	2.52 (1.85, 3.45)	1
RBC transfusion (OR (95% CrI))			
DC	1	0.63 (0.31, 0.96)	0.69 (0.38, 1.54)
MDC	2.24 (0.67, 2.85)	1	0.78 (0.51, 2.44)
MDCI	4.69 (2.79, 7.87)	2.64 (1.43, 4.05)	1
PLT transfusion (OR (95% CrI))			
DC	1	0.57 (0.32, 0.63)	0.77 (0.54, 1.63)
MDC	1.25 (1.03, 2.18)	1	0.75 (0.38, 1.75)
MDCI	4.41 (3.14, 5.17)	1.64 (1.03, 3.15)	1
G-CSF (OR (95% CrI))			
DC	1	0.36 (0.22, 0.85)	0.71 (0.34, 1.57)
MDC	1.43 (0.74, 3.10)	1	0.81 (0.62, 1.79)
MDCI	3.69 (2.72, 5.87)	2.94 (1.85, 3.74)	1
Hospitalization (OR (95% CrI))			
DC	1	0.46 (0.42, 0.76)	0.69 (0.44, 2.17)
MDC	1.10 (0.68, 2.85)	1	0.76 (0.31, 1.54)
MDCI	5.64 (2.92, 7.52)	1.38 (0.26, 3.25)	1

*HR* hazard ratio, *OR* odds ratio, *95% CrI* 95% credible intervals, *RBC* red blood cell, *PLT* platelet, *G-CSF* granulocyte colony-stimulating factor, *DC* doxorubicin + cisplatin, *MDC* methotrexate + doxorubicin + cisplatin, *MDCI* methotrexate + doxorubicin + cisplatin + ifosfamide

**Table 3:** Rankings based on simulations

Endpoints	Ranks	DC	MDC	MDCI
Overall survival (HR (95% CrI))	Rank 1	0.07	0.20	0.73
	Rank 2	0.21	0.67	0.13
	Rank 3	0.72	0.13	0.14
Progression-free survival (HR (95% CrI))	Rank 1	0.18	0.30	0.52
	Rank 2	0.23	0.53	0.24
	Rank 3	0.59	0.17	0.24
Total severe events (OR (95% CrI))	Rank 1	0.75	0.11	0.14
	Rank 2	0.20	0.68	0.12
	Rank 3	0.05	0.21	0.74
Leukopenia (OR (95% CrI))	Rank 1	0.69	0.12	0.18
	Rank 2	0.21	0.69	0.10
	Rank 3	0.10	0.18	0.72
Thrombocytopenia (OR (95% CrI))	Rank 1	0.72	0.14	0.14
	Rank 2	0.20	0.73	0.07
	Rank 3	0.07	0.13	0.79
RBC transfusion (OR (95% CrI))	Rank 1	0.55	0.31	0.14
	Rank 2	0.30	0.43	0.27
	Rank 3	0.15	0.25	0.60
PLT transfusion (OR (95% CrI))	Rank 1	0.63	0.20	0.17
	Rank 2	0.19	0.58	0.22
	Rank 3	0.19	0.21	0.59
G-CSF (OR (95% CrI))	Rank 1	0.71	0.12	0.17
	Rank 2	0.22	0.67	0.11
	Rank 3	0.07	0.20	0.73
Hospitalization (OR (95% CrI))	Rank 1	0.74	0.10	0.16
	Rank 2	0.21	0.69	0.10
	Rank 3	0.05	0.21	0.74

*HR* hazard ratio, *OR* odds ratio, *95% CrI* 95% credible intervals, *RBC* red blood cell, *PLT* platelet, *G-CSF* granulocyte colony-stimulating factor, *DC* doxorubicin + cisplatin, *MDC* methotrexate + doxorubicin + cisplatin, *MDCI* methotrexate + doxorubicin + cisplatin + ifosfamide

### Network meta-analysis of progression-free survival

As for PFS, there were 5 studies with 1249 patients reported the HR value or survival curve. The combined results revealed that MDCI had a clearly longer progression-free survival time than that of DC [MDCI: HR = 0.88, 95% CrI (0.46, 0.98)]. No statistical differences were found in MDC and DC. The network meta-analysis results were illustrated in Table 2. The probabilities of rank plot were as follows: MDCI ranked first (52.43%), MDC rank second (53.14%), and DC ranked third (62.77%). The related ranking results were presented in Fig. 4 b and Table 3.

### Network meta-analysis of total severe adverse events

There were 4 studies with 951 patients described the total number of severe adverse events. The pooled results showed that MDCI was associated with a greater total severe AEs than DC [MDCI: OR = 4.69, 95% CrI (2.79, 7.87)]. And no significant difference was found between MDC and DC. The analysis results were presented in Table 2. Furthermore, the rank results showed that DC ranked first (75.43%), MDC ranked second (68.17%), and MDCI ranked third (74.02%). The results of rank plot analysis were illustrated in Fig. 4 c and Table 3.

### Network meta-analysis of severe adverse events

As for the reported severe AEs in included studies, 6 common events including leukopenia, thrombocytopenia, RBC transfusion, PLT transfusion, G-CSF transfusion, and hospitalization were taken into analysis. The network analysis results are presented in Table 2. Compared with DC, MDCI showed obviously less AEs of leukopenia, thrombocytopenia, RBC transfusion, PLT transfusion, G-CSF transfusion, and hospitalization. In addition, MDC was associated with more severe adverse events such as thrombocytopenia and PLT transfusion than DC. Leukopenia is the most common adverse events in the MDCI regimen. No statistical difference was found in the rest of the comparisons.

### Consistency and convergence analysis

Node-splitting analysis was applied to evaluate inconsistency by comparing the differences between direct and indirect evidence. No significant inconsistency was detected among the various treatments, with the *P* value being less than 0.05. This meant that the consistency model was reliable. In addition, the potential scale reduction factor (PSRF) was limited to 1, and our study achieved good convergence efficiency. The publication bias of all outcomes was analysis by funnel plot, and no obvious publish bias was detected.

## Discussion

Osteosarcoma, a rare type of sarcoma, differs greatly in its pathogenesis and biological behavior [25]. About 20–30% of patients present with metastatic osteosarcoma, most commonly to the lungs, lymph nodes, or other bones [26, 27]. Nowadays, standard treatment of high-grade non-metastatic and metastatic osteosarcoma involves neoadjuvant multiagent chemotherapy followed by surgical resection of the lesions and adjuvant multiagent sequential chemotherapy [28, 29]. As the survival rate increases from 20 to 80%, chemotherapy for treatment of osteosarcoma was demonstrated to be effective [30]. The efficacy of frontline chemotherapy agents (high-dose methotrexate, doxorubicin, cisplatin, and ifosfamide in the treatment of osteosarcoma) has been investigated; however, results are inconsistent.

This study is a network meta-analysis with the objective of assessing the efficacy and safety of first-line chemotherapeutic agents for osteosarcoma. In our study, 7 high-quality RCTs with 1532 patients were included and analyzed. The efficacy was assessed by the outcomes of OS, PFS. In addition, we evaluated the safety through total number of severe AEs and several common related AEs. Based on the results of the analysis, we found that the regimen MDCI could significantly increase OS, PFS, and severe AEs compared with DC. Furthermore, MDCI had a significant lower hazard risk of OS than MDC. While, there was no significant difference in PFS for MDCI and MDC. The efficacy of MDCI has been proven in the trails of Ferrari, Link and Meyers [18, 21, 23]. According to the ranking results, MDCI ranked first in OS and PFS. Regarding safety outcomes, however, MDCI was ranked third after MDC and DC. There were no direct comparisons of MDCI and DC. The relationship between MDCI and DC, however, was obtained by indirect comparisons in our study. According to the results, methotrexate and ifosfamide may contribute to the chemotherapy approach.

Methotrexate (MTX) seems to be one of the most active agents, however, at the moment, it is not clear about the absolute role of it in multidrug chemotherapy [10, 11]. In the literature, the use of MTX in osteosarcoma from the initial single-agent to the complex combination regimens can be found. Rosen et al. [31] measured the tumor response at the time of surgery to assess the efficacy of preoperative MTX. A good histologic response with > 90% tumor necrosis was observed in 25 of 32 (78%) patients with primary disease after four weekly doses of 8–12 g/m^2^ [31, 32]. The response rate of the combination of MTX has not been clearly defined [33]. Bramwell et al. [19] conducted a RCT comparing 2 chemotherapy schemes: DC versus high dose MTX with reduced dose doxorubicin and cisplatin. PFS was inferior in patients who received the 3-drug arm but OS was not significantly different between both treatment arms. Bacci et al. [34] demonstrated that patients receiving MTX had a significant lower hazard risk of overall survival rates at 5 years. These results confirmed the significant survival benefits of methotrexate-based chemotherapy for osteosarcoma. These findings are similar to our network meta-analysis results.

Ifosfamide, an alkylating agent, works by disrupting the tumor cell’s microtubule dynamics, was introduced approximately 20 years later [35]. As an analog of cyclophosphamide, it is highly active in the treatment of osteosarcoma [36]. In a phase II study of ifosfamide in the treatment of recurrent sarcomas in young people concluded by Magrath et al. [37], patients who experienced relapse after standard therapies have shown remarkable responses when they were subjected to ifosfamide-based chemotherapy. In the meta-analysis of Fan et al. [38], ifosfamide-based chemotherapy reduced the risk of death in patients with osteosarcoma by 17% (HR = 0.83, 95% CrI 0.70, 0.99; *P* = 0.034). Meta-analysis, the highest level of evidence, showed the efficacy of ifosfamide for osteosarcoma. Therefore, the role of ifosfamide in the MDCI regimen may contribute to the chemotherapy advantage compared with MDC.

Even though multidrug regimens, such as MDCI and MDC, had a better effect on prolonging the PFS and OS of osteosarcoma patients, it should be recognized the serious adverse effects [39]. According to our results, that MDCI was associated with a greater total severe AEs than DC [MDCI: OR = 4.69, 95% CrI (2.79, 7.87)]. Serious adverse effects such as leukopenia and thrombocytopenia will affect the application of the regimens and even the quality of life of patients [40, 41]. The reduction of adverse events is a direction for the wide application of multidrug chemotherapy [42, 43].

In recent years, studies co-sponsored by European and American Osteosarcoma Studies (EURAMOS), Cooperative Osteosarcoma Study Group (COSS), European Osteosarcoma Intergroup (EOI), and Scandinavian Sarcoma Group (SSG) have yielded encouraging results [44–46]. Smeland et al. [44] enrolled 2186 patients over a 6-year period and evaluated their differences after receiving MDC chemotherapy. As their conclusions show, nearly 4 out of every 5 patients with non-metastatic osteosarcoma who have all disease resected and MDC finished are alive 5 years later, and the risk of relapse appears to decrease over time. Since their study did not directly compare MDC and MDCI, their data was not suitable for inclusion in our work.

Neoadjuvant and adjuvant chemotherapy combined with resection has become the basic strategy for the treatment of osteosarcoma [47]. Neoadjuvant chemotherapy includes the addition of chemotherapy drugs before resection, and this regimen has the following advantages: (1) It can control the primary tumor and reduce the chance of surgical tumor invasion. (2) It can eliminate micrometastasis early and avoid metastasis caused by delayed surgery or low resistance. (3) It can evaluate the effect of chemotherapy and guide comprehensive treatment after surgery. (4) It can assess the prognosis earlier. Although the results of RCTs suggested no significant effect on the outcome of patients when comparing preoperative chemotherapy to postoperative chemotherapy [48], neoadjuvant chemotherapy for limb salvage and the surgical process is still worthy of clinical application. Independent comparisons and discussions of neoadjuvant chemotherapy and adjuvant chemotherapy were not mentioned in our study. As we know, the resistance of chemotherapy does not change with or without surgery [49]. A large number of studies have confirmed that MDCI shows the same efficacy in neoadjuvant chemotherapy and adjuvant chemotherapy [24, 50].

The extent of histologic response may have an impact on the EFS outcome. Patients with > 90% tumor necrosis were classified as good responders (GRs), whereas patients with < 90% were defined as poor responders (PRs). In the study conducted by Ferrari et al. [23], 5-year EFS was 69% (95% CrI, 60 to 78%) for GRs and 52% (95% CrI, 44 to 61%) for PRs. The purpose of our study was to briefly evaluate the efficacy of four chemotherapeutic drugs (high-dose methotrexate, cisplatin, doxorubicin, and ifosfamide) prescribed in the NCNC Guidelines for patients with osteosarcoma. The regrouping and discussion based on the histologic response of patients were not covered, so we can draw a conclusion that is applicable to primary hospitals (inability to evaluate tumor necrosis after surgery), especially in developing countries like China [51]. We hope that our study with the statistical advantage of meta-analysis can provide a concise guide for chemotherapy of osteosarcoma.

There were methodological strengths in our study as follows: (1) comprehensive retrieval strategy was applied to reduce the risk of publication bias; (2) the application of rank plot can distinguish subtle differences among all chemotherapy agents; and (3) the study was the first comparison of direct and indirect approaches, which incorporated all available data to evaluate the interventions more precisely.

Nevertheless, our meta-analysis does have certain limitations. First of all, no direct comparison between MDCI and DC was found and included, which may have caused inaccurate results in our analysis. In order to avoid interference with other agents, our analysis only included trials that their regimen is any combination of methotrexate, doxorubicin, cisplatin, and ifosfamide. Therefore, many RCTs containing irrelevant agents such as vincristine and actinomycin-D were excluded. Secondly, for some included RCTs, the detailed blind methods and allocation concealment were not described which could affect the validity for overall findings. Thirdly, we just analyzed nine types of outcomes from the variable endpoints. This could miss several necessary outcomes and affect the whole analysis results.

## Conclusion

MDCI showed its superiority among all chemotherapeutic regimens in relation to efficacy and safety, followed by MDC. In addition, MDCI was associated with an increased risk of AEs. According to our analysis, DC was less effective but safer for MDC and MDCI. Therefore, we recommended MDCI as the optimal choice for osteosarcoma. However, considering limitations of our network meta-analysis, additional high-quality studies are needed for further evaluation.

## Data Availability

All data generated or analyzed during this study are included in this published article.

## Abbreviations

95% CrI: 95% credible intervals
C: Cisplatin
D: Doxorubicin
DC: Doxorubicin + cisplatin
G-CSF: Granulocyte colony-stimulating factor
HR: Hazard ratio
I: Ifosfamide
M: Methotrexate
MDC: Methotrexate + doxorubicin + cisplatin
MDCI: Methotrexate + doxorubicin + cisplatin + ifosfamide
OR: Odds ratio
PLT: Platelet
RBC: Red blood cell
